# Cervical Esophageal Characteristics in Smokers Versus Non-Smokers: An Ultrasonographic Comparative Analysis

**DOI:** 10.3390/diagnostics16091343

**Published:** 2026-04-29

**Authors:** Muhammed J. Alsaadi, Abdulrahman M. Alfuraih

**Affiliations:** Radiology and Medical Imaging Department, College of Applied Medical Sciences, Prince Sattam bin Abdulaziz University, Kharj 11942, Saudi Arabia; m.alsaadi@psau.edu.sa

**Keywords:** cervical, esophagus, wall thickness, morphology, smokers, ultrasound, cancer

## Abstract

**Background/Objective**: Smoking is known to be associated with reflux-related mucosal damage and deleterious esophageal outcomes, yet no non-invasive imaging biomarkers of smoking-induced esophageal remodeling have been identified. We aimed to compare cervical esophageal ultrasound morphology between habitual smokers and non-smokers, in terms of esophageal wall thickness, number of sonographically discernable wall layers, and esophageal diameter, and investigate whether smoking is an independent predictor of these findings. **Methods**: In this cross-sectional study, 60 participants (30 smokers, 30 non-smokers) underwent high-resolution B-mode ultrasound of the cervical esophagus. Examinations were performed in transverse and longitudinal planes. Outcomes included esophageal wall thickness (mm), number of discernible wall layers, and esophageal diameters in transverse and longitudinal planes. Group comparisons used independent *t*-tests and chi-square tests. Multiple linear regression assessed independent associations with smoking status (adjusting for age and weight). Within smokers, Pearson correlation evaluated relationships between smoking duration and ultrasound outcomes; exploratory subgroup analyses compared smoking modalities. **Results**: Smokers were older and had higher weight and BMI than non-smokers. Compared with non-smokers, smokers had greater wall thickness (3.06 vs 2.61 mm), more discernible wall layers (5.03 vs 3.60), and larger transverse (11.68 vs 7.87 mm) and longitudinal (12.90 vs 8.26 mm) diameters (all *p* < 0.001). In regression analysis, smoking status independently predicted wall thickness (B = 0.411 mm, 95% CI 0.243–0.578; *p* < 0.001). Smoking duration showed significant correlations with the number of visible layers (r = 0.82; *p* < 0.001) and wall thickness (r = 0.42; *p* = 0.021). **Conclusions**: High-frequency ultrasound detected significant differences in cervical esophageal morphology between smokers and non-smokers. Smoking was independently associated with differences in the diameter, thickness, and number of visible layers of the cervical esophagus. Further studies with larger sample sizes, improved exposure assessment, and use of reference standards are needed.

## 1. Introduction

Cigarette smoking is a known risk factor for physiologic mechanisms of esophageal injury. Experimental and clinical manometric studies have shown that cigarette smoking is associated with lower esophageal sphincter (LES) pressure and a higher rate of reflux episodes while smoking, offering a direct mechanism for increased acid exposure of the esophageal mucosa [1,2]. More broadly, gastroesophageal reflux disease (GERD) risk factors emphasize that tobacco can facilitate reflux not only via LES effects, but also by impairing protective mechanisms such as bicarbonate-rich salivary buffering and esophageal clearance, thereby promoting an environment of repeated mucosal insult [2]. Over time, recurrent reflux-related exposure and smoke-related epithelial injury may sustain chronic inflammation and remodeling responses, offering a biological rationale for why smoking could be associated with measurable structural differences in the esophageal wall.

From an imaging perspective, “wall thickening” is a recognized downstream manifestation of esophageal inflammation across modalities, supporting the plausibility of thickness as a quantitative marker of mucosal–submucosal injury and remodeling. In CT-based series of clinically diagnosed esophagitis, circumferential esophageal wall thickening (often using a ≥5 mm cutoff) is observed, and longer segments of mural thickening (with or without a target sign) are read by radiologists as evidence of esophagitis in the appropriate clinical setting [3]. While thickening is not disease-specific, the consistent appearance of mural thickening in inflammatory diseases of the esophagus provides an anatomic and pathophysiologic linkage between exposure-related injury (i.e., smoking-related reflux and reflux-related epithelial injury) and a structural endpoint that can be evaluated using ultrasound-based measures.

Ultrasound is ideally suited to the evaluation of such endpoints, as it is non-invasive, does not expose the subject to ionizing radiation, can be easily repeated, and has excellent spatial resolution in superficial structures. Foundational sonographic descriptions have defined the normal cervical esophagus, its expected relationship to the trachea, and its transverse “rosette-like” appearance when the lumen is collapsed, while subsequent technical reports have proposed modified, high-resolution techniques that optimize wall delineation and measurement reproducibility using linear transducers [4,5]. Pediatric studies have further established the feasibility of sonographic assessment of the cervical and thoracic segments of the esophagus, and recent findings indicate that sonographers achieve a quantifiable learning curve for identifying the cervical esophagus on ultrasound in healthy volunteers, supporting its use for standardized acquisition of ultrasound images [6,7].

The cervical esophagus is the only esophageal segment accessible to transcutaneous high-frequency ultrasound without the need for invasive endoscopic access, making it uniquely suited for non-invasive research phenotyping in healthy volunteers. Second, while distal mucosal injury from acid refluxate is well-characterized, the systemic effects of tobacco smoke—including LES dysfunction, impaired esophageal peristaltic clearance, and pro-inflammatory circulating mediators—are not anatomically restricted to the distal esophagus [6,7]. Chronic reflux burden and systemic mucosal insult could plausibly produce wall remodeling across the entire esophagus, including the cervical segment. Third, prior ultrasound and endoscopic ultrasound (EUS) studies confirm that wall layer stratification and thickness changes are measurable features of inflammatory and exposure-related esophageal disease, providing justification for applying quantitative wall characterization at the cervical level [8,9].

Previous ultrasound studies on the esophagus have examined two domains that are informative, yet do not address a key question for this effect of smoking exposure. First, disease-cohort studies—particularly in eosinophilic esophagitis (EoE) and GERD—have evaluated wall thickness and related morphologic metrics using conventional ultrasound or EUS, sometimes linking thickening (including mucosal–submucosal components) to symptom burden such as dysphagia, while also highlighting the limits of thickness alone as a standalone diagnostic discriminator [10,11,12,13,14]. Second, ultrasound (including point-of-care applications) and EUS have been explored in the context of esophageal malignancy detection and staging, where wall layer integrity and depth of invasion are central concepts [15,16,17,18]. Across these studies, smoking is often treated as a clinical risk factor rather than an imaging covariate, and few studies have examined smoking status as a primary determinant of ultrasound-measured esophageal wall characteristics.

Accordingly, the present study used high-frequency transcutaneous ultrasound to compare cervical esophageal morphology between habitual smokers and non-smokers. Specifically, the study aimed to quantify differences in esophageal wall thickness and wall layer conspicuity (number of discernible wall layers), to evaluate group differences in transverse and longitudinal esophageal diameters, and to assess whether smoking status remained independently associated with these ultrasound markers.

## 2. Materials and Methods

### 2.1. Study Design

A cross-sectional study was conducted to assess cervical esophageal wall characteristics by ultrasound. A quantitative research approach was employed for objective assessment of esophageal wall thickness and morphology in two groups of healthy, non-smoker and smoker participants. All imaging was conducted under controlled conditions in a clinical setting at Prince Sattam bin Abdulaziz University, using conventional B-mode ultrasound imaging techniques. The study was approved by the local ethical committee (IRB approval: REC-HSD-117-225; date: 14 September 2025). Informed consent was provided by all participants.

### 2.2. Primary and Secondary Outcomes

The primary outcome of this study was cervical esophageal wall thickness (mm), selected a priori as the most clinically relevant morphological marker of mucosal–submucosal remodeling. Secondary outcomes were the number of sonographically discernible wall layers and esophageal diameter in the transverse and longitudinal planes. Comparisons of ultrasound outcomes between groups were the primary analyses. Subgroup analyses by smoking modality (cigarettes, vaporizer, combined) were pre-specified as exploratory.

### 2.3. Participant Selection

Sixty participants (*n* = 60) were prospectively assigned to two groups of equal size (30 each) based on smoking status (30 smokers, 30 non-smokers). The following variable measurements were obtained: esophageal wall thickness (mm), number of visualized wall layers, and cervical positioning (midline: Yes/No). All measurements were taken in transverse (TVS) and longitudinal (LUS) imaging planes.

Inclusion criteria consisted of male and female participants aged 15 years or older and university students or employees at Prince Sattam bin Abdulaziz University, with informed written consent obtained. Smokers were identified as persons who had smoked continuously for ≥5 years (of any form, including conventional cigarettes, electronic devices, or shisha). Exclusion criteria involved participants with a history of any esophageal symptoms disorder, pregnant women, and individuals unable or unwilling to provide informed consent.

### 2.4. Ultrasound Protocol

**Figure 1:**
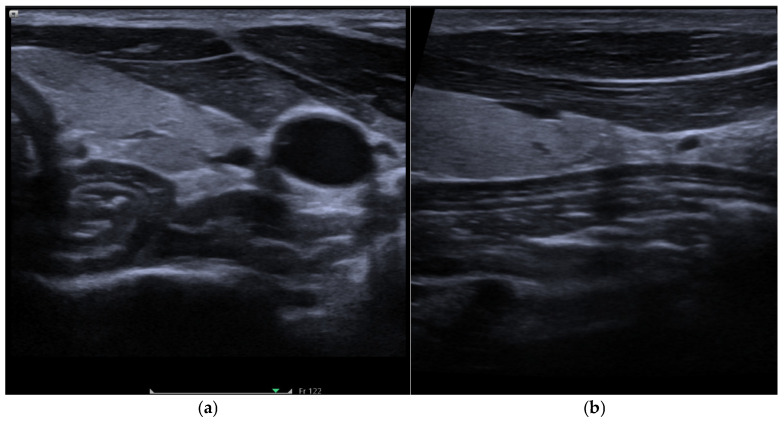
Representative B-mode ultrasound images of the cervical esophagus in (**a**) transverse view and (**b**) longitudinal view.

Ultrasound imaging was performed using a Siemens ACUSON Juniper system (Siemens Healthineers, Munich, Germany) with a high-frequency 11L4 MHz linear transducer. The cervical esophagus was scanned in transverse and longitudinal planes (Figure 1). The following measurements were taken: (i) cervical position of the esophagus (right, left, or midline), (ii) number of visualization layers, (iii) wall thickness in millimeters, and (iv) imaging plane with optimal viewing of maximum stratification of the wall structure. The scanning was performed by a consultant, who had >25 years of practical experience in medical ultrasonography. The sonographer was blinded to all participant characteristics, including smoking status.

All measurements were performed with the participant in the supine position with the neck in a neutral, slightly extended position. Images were acquired at rest, with participants instructed to refrain from swallowing during image acquisition to minimize dynamic luminal caliber variability. Esophageal wall thickness was measured from the anterior esophageal wall, as this is the most reliably and reproducibly visualized surface on transcutaneous imaging. Wall layer conspicuity was assessed using a standardized gain setting and focal zone depth fixed at the level of the cervical esophagus. Discernible wall layers were defined as discrete, alternating hyperechoic–hypoechoic bands visible under these standardized conditions, consistent with established high-frequency ultrasound and EUS layer nomenclature (representing combinations of mucosa, submucosa, muscularis propria, and adventitia/serosa) [8,9].

### 2.5. Statistical Analysis

Statistical analyses were performed using JASP software (version 0.95). Normality was determined using the Shapiro–Wilk test and histogram inspection. Group comparisons for continuous variables were performed using an independent *t*-test (with Welch’s correction for unequal variances); effect sizes were estimated using Cohen’s d. Group comparisons for categorical variables were performed using chi-square tests.

Pearson’s correlation coefficients were tested for associations between smoking duration and wall thickness only in smoker group participants. Analysis of variance for sub-categories of smoking type (vaporizers, cigarettes, vaporizers and cigarettes) was performed using one-way ANOVA for continuous outcome variables. If significance was achieved, post hoc tests (Tukey’s HSD) were performed.

Multiple linear regression analyses were performed for smoker/non-smoker status of the groups. They included the following confounding factors: number of smoking years, age at enrolment and current age, and sex. Values for variance inflation factor (VIF; <5) were tested for multicollinearity influences upon model fit. Model fit was reported as R^2^, adjusted R^2^ and F-statistic values, and condition number. Residuals were examined for normality using Shapiro–Wilk tests; these interpretations were valid even with moderate violations (*n* = 60). Weight was used as the body size covariate in the primary regression models rather than BMI, as height did not differ significantly between groups (*p* = 0.070) and weight and BMI were highly correlated in this sample (r ≈ 0.98), introducing multicollinearity if both were included. A *p*-value threshold of <0.05 was set for tests of significance.

No formal a priori power calculation was conducted; sample size was determined based on participant availability and feasibility. Post hoc analysis indicated that for the primary outcome (wall thickness, Cohen’s d = 1.82), the achieved sample size of *n* = 30 per group provided >99% statistical power at α = 0.05 (two-tailed). For the smallest observed effect (BMI difference, d = 0.88), power exceeded 80% at this sample size. These retrospective estimates suggest adequate power for the large effects observed; however, the absence of a priori justification is acknowledged as a methodological limitation.

## 3. Results

Sixty male participants were included (30 non-smokers and 30 smokers). Smokers were older and had higher body weight and BMI than non-smokers (≈14% older, 18% higher body weight, and 14% higher BMI; Table 1), while height was almost similar between groups. Among smokers, the mean duration of smoking was 7.93 ± 2.48 years; 6.7% used vaporizers only (*n* = 2), 30.0% cigarettes only (*n* = 9), and 63.3% both vaporizers and cigarettes (*n* = 19).

**Figure 2:**
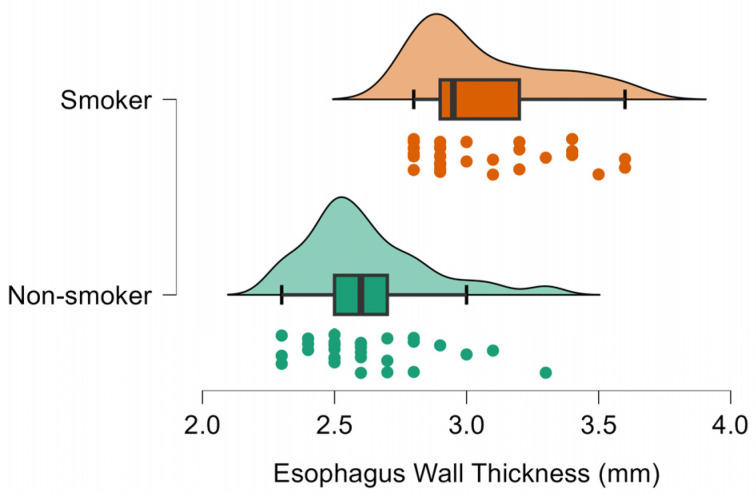
Raincloud plot of cervical esophageal wall thickness (mm) in smokers and non-smokers. Smokers show markedly thicker walls (mean ≈ 3.1 mm) compared with non-smokers (≈2.6 mm).

**Figure 3:**
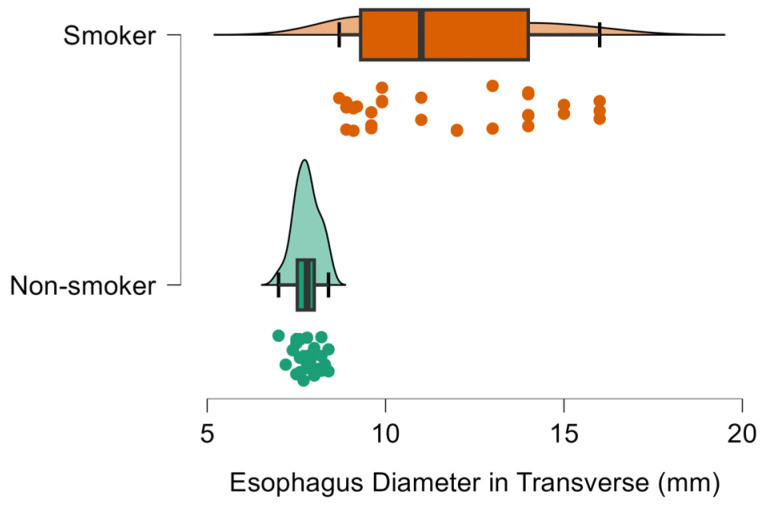
Raincloud plot of cervical esophageal transverse diameter (mm) in smokers and non-smokers. Smokers have substantially larger transverse diameters (mean ≈ 11.7 mm) than non-smokers (≈7.8 mm).

**Figure 4:**
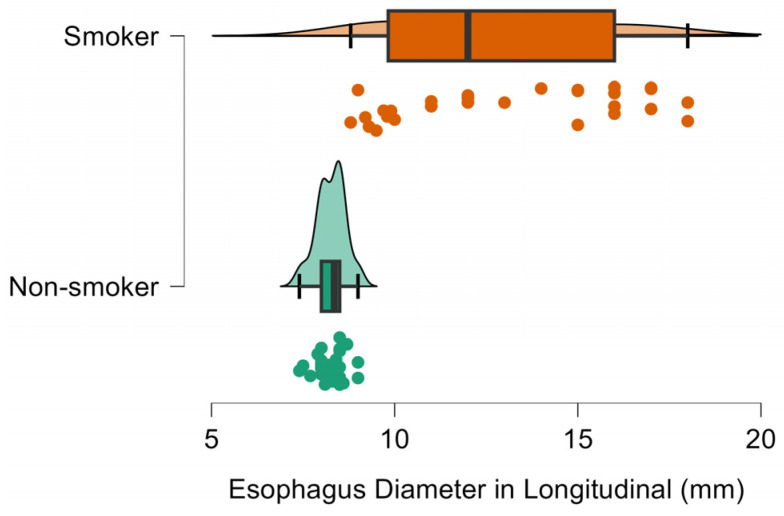
Raincloud plot of cervical esophageal longitudinal diameter (mm) in smokers and non-smokers. Longitudinal diameters are greater in smokers (mean ≈ 12.9 mm) than in non-smokers (≈8.3 mm).

Compared with non-smokers, smokers had thicker cervical esophageal walls (0.31 vs 0.26 cm; ≈19% increase; Figure 2), more discernible wall layers (5.03 vs 3.60; ≈40% increase), and larger esophageal diameters in both transverse (1.17 vs 0.78 cm; ≈50% increase; Figure 3) and longitudinal planes (1.29 vs 0.83 cm; ≈55% increase; Figure 4), all *p* < 0.001 (Table 1).

Separate multiple linear regression models were constructed for the four main ultrasound outcomes (wall thickness, number of wall layers, and esophageal diameters in the transverse and longitudinal planes). The results are shown in Table 2. For esophageal wall thickness, the multiple linear regression model including age, weight, and smoking status (smoker vs non-smoker) was statistically significant and explained 48.0% of the variance (R = 0.693, R^2^ = 0.480, adjusted R^2^ = 0.452, F (3.56) = 17.23, *p* < 0.001). After adjustment for age and weight, smoking remained the only independent predictor: smokers had on average 0.41 mm thicker esophageal walls than non-smokers (B = 0.411, 95% CI 0.243–0.578 mm, *p* < 0.001), whereas age (B = 0.014 mm/year, *p* = 0.201) and weight (B = −0.0009 mm/kg, *p* = 0.738) were not significantly associated with wall thickness.

In the remaining models, smoking status consistently emerged as the main independent predictor of esophageal morphology. For the number of discernible wall layers, the model including age, weight, and smoking status explained 59.9% of the variance (R^2^ = 0.599, adjusted R^2^ = 0.578, F (3.56) = 27.94, *p* < 0.001). Smokers had on average 1.33 more discernible layers than non-smokers (B = 1.325, 95% CI 0.88–1.77, *p* < 0.001), corresponding to an increase of about 40% relative to non-smokers (mean 3.6 vs 5.0 layers). For the transverse diameter, the model explained 59.6% of the variance (R^2^ = 0.596, adjusted R^2^ = 0.574, F (3.56) = 27.55, *p* < 0.001), and smokers had 3.9 mm larger diameters than non-smokers (B = 3.923, 95% CI 2.73–5.11 mm, *p* < 0.001), representing an increase of approximately 50% (mean 7.8 vs 11.7 mm). Similarly, for the longitudinal diameter, the model accounted for 57.4% of the variance (R^2^ = 0.574, adjusted R^2^ = 0.551, F (3.56) = 25.18, *p* < 0.001), and smokers showed 4.7 mm greater longitudinal diameters (B = 4.660, 95% CI 3.19–6.13 mm, *p* < 0.001), which corresponds to an increase of around 56% compared with non-smokers (mean 8.3 vs 12.9 mm).

Among smokers, smoking duration showed a strong positive correlation with the number of discernible wall layers (r = 0.82, *p* < 0.001) and a moderate positive correlation with wall thickness (r = 0.42, *p* = 0.021) but was not significantly related to esophageal diameters in either plane.

Within smokers, subgroup analysis by smoking type (cigarettes, vaporizers, combined vaporizers and cigarettes) showed no significant differences in wall thickness or esophageal diameters (wall thickness F (2.27) = 0.60, *p* = 0.557; transverse diameter F (2.27) = 1.64, *p* = 0.213; longitudinal diameter F (2.27) = 1.60, *p* = 0.220; Table 3). In contrast, the number of discernible wall layers differed across smoking types (F (2.27) = 4.36, *p* = 0.023), with the vaporizers and cigarettes combined users showing more layers on average (5.32 vs 4.56 and 4.50), corresponding to roughly 17–18% higher layering compared with cigarettes-only and vaporizers-only smokers. These subgroup findings are purely exploratory and must be interpreted with extreme caution. The vaporizer-only subgroup (*n* = 2) is wholly insufficient for statistical inference; any apparent differences in this group should not be interpreted as representing the effect of vaporizers. The significant ANOVA result for number of wall layers (*p* = 0.023) is similarly limited by the extreme group size imbalance and should not be used to draw conclusions about smoking modality effects without replication in adequately powered studies.

## 4. Discussion

In this ultrasound study of the cervical esophagus, smoking was associated with consistent and statistically strong structural differences compared with non-smoking controls. Smokers demonstrated greater esophageal wall thickness, a higher number of discernible wall layers, and larger esophageal diameters in both transverse and longitudinal planes, with large effect sizes across all major ultrasound outcomes. Additionally, multivariable modeling indicated that smoking status remained the dominant independent predictor of wall thickness and wall layer conspicuity after accounting for demographic differences between groups, suggesting that the observed morphological changes are not readily explained by age or body size alone. Within the smoker group, longer smoking duration correlated with the number of discernible layers and moderately with wall thickness, suggesting a dose–response signal that supports the plausibility of cumulative exposure-related esophageal wall remodeling.

The current findings are broadly consistent with limited literature showing that esophageal wall thickening is a measurable feature of chronic inflammatory esophageal disease, particularly when assessed with endoscopic or high-frequency techniques. For example, using EUS, Fox et al. reported a significant increase in total distal esophageal wall thickness in children with eosinophilic esophagitis compared with controls, with thickening evident within specific layers (combined mucosa–submucosa and muscularis propria [19]. In adults, Wong et al. similarly demonstrated that distal esophageal wall thickening, especially within the submucosa, distinguished eosinophilic esophagitis from reflux disease and correlated with clinical severity (dysphagia) and disease duration, supporting the concept that chronic exposure and remodeling can manifest as increased wall thickness [11]. While pediatric EUS work has suggested that thickness measurements alone may have limited discriminative performance for diagnosing eosinophilic esophagitis in small cohorts, these studies nonetheless provide important precedent that ultrasound can quantify layer-specific wall remodeling in vivo [12]. Additional conventional ultrasound data in children have also shown that cervical esophageal wall thickness can differ across inflammatory phenotypes (eosinophilic esophagitis vs gastroesophageal reflux disease), however, with only moderate diagnostic accuracy, highlighting both the feasibility and the need for carefully defined imaging targets and clinical context [10]. These observations support the biological plausibility that the thicker cervical esophageal walls observed in smokers in the present study may reflect chronic exposure-related remodeling, even though the underlying triggers and the anatomic segment examined differ from the EoE/GERD literature.

Measurement precision and clinical significance of the wall thickness difference. The observed mean wall thickness difference between groups was 0.45 mm (3.06 vs. 2.61 mm; Cohen’s d = 1.82). While this difference is statistically significant and accompanied by a large effect size, its clinical significance warrants careful consideration. The 11L4 MHz linear probe has an axial resolution of approximately 0.15–0.20 mm at cervical esophageal depths, meaning the observed mean difference acceptably exceeds the physical resolution limit of the transducer. However, the between-group standard deviation (≈0.25 mm) approaches the magnitude of the effect, and in the absence of intra- and inter-observer testing, the proportion of the observed difference attributable to true structural change versus physiological variability cannot be precisely determined. Comparison with prior sonographic normal-range data [4,5] suggests that our non-smoker values (2.61 ± 0.23 mm) are consistent with published cervical esophageal wall thickness measurements in healthy adults, lending confidence to the baseline reference values.

The substantially larger esophageal diameters observed in smokers across both transverse and longitudinal planes may reflect complementary changes in wall structure and esophageal function rather than wall thickening alone. Conventional sonographic studies, particularly in pediatric populations, have shown that the cervical and thoracic esophagus can be assessed non-invasively for caliber-related measures and dynamic changes during swallowing, and that esophageal caliber/distensibility indices can vary across reflux-related phenotypes [6,20]. In the current cohort, increased diameters may therefore plausibly reflect increased wall bulk leading to increased measured outer diameter in combination with reflux-related functional changes influencing the resting caliber of the cervical esophagus. Cross-sectional imaging studies have also demonstrated that inflammatory conditions of the esophagus may be associated with detectable changes in mural thickening and in luminal caliber [3]. This provides more basis for considering increases in diameter as a manifestation of the exposure-related remodeling rather than as an isolated geometric change. In comparison with published sonographic normal ranges for the cervical esophagus [4,5], our non-smoker diameter values fall within expected ranges for adults imaged at rest, supporting the validity of the baseline measurements. The consistently significant regression coefficients for smoking status (approximately 4–5 mm larger diameters in smokers, *p* < 0.001) after adjustment for age and weight suggest that the differences are not fully explained by demographic factors. Nonetheless, the magnitude of the difference in a young, asymptomatic population without endoscopic or functional correlates should be interpreted cautiously, and replication in a larger sample with standardized post-swallow interval timing is recommended.

The conducted multivariable analyses also help to reinforce the conclusion that smoking is independently associated with this remodeling of the cervical esophagus, as smoking status remained the overall primary predictor of wall thickness and wall stratification after correction for age and body weight. The observed age difference between groups (14.3% higher mean age in smokers) and the significant BMI difference (14.4% higher in smokers) were potential confounders that warranted careful adjustment. Age-related changes in esophageal wall compliance and BMI-related increases in intra-abdominal pressure and reflux susceptibility could theoretically contribute to wall morphology differences independent of smoking. However, in the primary regression model, neither age (*p* = 0.201) nor weight (*p* = 0.738) were significantly associated with wall thickness after adjustment for smoking status. This finding does not eliminate the possibility of residual confounding—particularly from unmeasured variables such as GERD symptom burden, physical activity, or dietary factors—but it does indicate that the group differences in esophageal wall morphology are not simply attributable to the age or body size differences between groups. This finding is clinically relevant in that these anthropomorphic characteristics could be expected to alter measurements of the esophageal wall in some contexts, especially when considering pediatric populations, in whom prediction models for anticipated EUS-derived thickness have previously included age and body size as predictors [21]. In the current cohort, however, neither age nor body weight was significantly associated with wall thickness once smoking status was controlled, highlighting that the visible difference between groups is not simply due to anticipated biometric variation. However, residual confounding cannot be completely excluded. For example, smokers did differ from non-smokers with regard to mean age and mean BMI, and the study design did not intend to perfectly match participants with regard to these factors. Future investigations may help to strengthen the likelihood of a causal inference through group matching or stratification (e.g., age- and BMI-matched cohorts), and by additionally quantifying additional exposures (e.g., pack years) or by use of other biomarkers while adhering to the imaging protocol that has been shown to be feasible for these evaluations for the cervical esophagus [5,7].

Within the smoker population, the visible dose–response effect does support the notion that the observable sonographic differences represent remodeling of the cervical esophageal wall as a result of tobacco exposure rather than binary remodeling-related changes resulting from having smoked at any point in time. This interpretation is also biologically plausible in that tobacco exposure is associated with persistent mucosal injury and inflammatory signals in the gastrointestinal tract [22], and in that experimental models of Barrett’s related injury have suggested that tobacco smoke exposure has the potential to potentiate damage-related molecular pathways [23] (particularly for injury caused by refluxate). General population-based studies have also noted that cigarette smoking is associated with increased rates of Barrett’s esophagus [24], and relatively elevated rates of esophageal cancer in high-risk groups [25], further highlighting the potential for this exposure to induce clinically important changes to the esophagus over time. CT imaging studies have also demonstrated that inflammatory conditions of the esophagus may be associated with mural thickening [3], thus providing a further basis for the potential interpretation of measurable wall thickness as a result of chronic injury. The lack of any meaningful relationship between duration of smoking and either plane diameters, however, suggests that wall thickness and layer conspicuity may be more sensitive indicators of cumulative exposure than diameter measurements in this asymptomatic population. Future studies should measure pack years, as well as correlate symptoms, in order to further evaluate this effect.

Subgroup analyses by smoking type did not demonstrate statistically significant differences in wall thickness or diameters across cigarettes-only, vaporizers-only, and dual-use groups; however, dual users showed a higher number of discernible wall layers. This pattern must be interpreted as entirely exploratory and the vaporizer-only group findings (*n* = 2) are insufficient for any inference about modality-specific effects. Nevertheless, the observation that “layer conspicuity” (rather than thickness) differed by smoking pattern is conceptually compatible with the idea that cumulative and/or combined exposures may preferentially influence mucosal–submucosal interface characteristics, which are the interfaces that determine how many layers can be resolved on high-frequency imaging, rather than uniformly increasing bulk thickness alone [8,9].

From a biological standpoint, dual exposure could plausibly amplify injury-related signaling pathways, particularly in the presence of reflux elements, as suggested by experimental data demonstrating that tobacco smoke can potentiate bile-related molecular responses in Barrett’s associated models [23], and by epidemiologic evidence linking smoking with increased risk of Barrett’s esophagus and downstream esophageal pathology [24,25]. Importantly, these mechanistic and epidemiologic links do not imply that the present ultrasound findings represent premalignant change; rather, they provide a plausible exposure–injury framework within which differences in sonographic wall stratification could emerge.

Future studies designed to directly compare smoking forms head-to-head with balanced group sizes, better exposure metrics (pack years, vaping frequency, hookah frequency), and correlates of symptoms are needed before any conclusions can be drawn regarding cervical esophageal effects of cigarettes versus vaporizers.

Overall, these findings indicate that high-frequency ultrasound assessment of the cervical esophagus may be a feasible and non-invasive method for phenotyping and potentially longitudinally quantitating exposure-based differences in cervical esophageal structure in vivo when endoscopic assessment is not practical or applicable for research screening. Previous studies using ultrasound have demonstrated consistent visualization of the cervical and thoracic esophagus with ultrasound and further specified the wall with standardized techniques to allow the quantification of wall thickness in normal and clinical applications [4,5,6,7]. Furthermore, ultrasound-based methodologies have also been utilized in phenotyping and functional studies related to reflux, confirming ultrasound’s emerging role as an adjunctive methodology in the assessment of esophageal disorders [20]. Concurrently, the endoscopic ultrasound (EUS) literature establishes clinically relevant cutoffs for wall layer and layer-specific thickening in inflammatory disease and neoplasia staging, thereby supporting the conceptual endpoints of wall layer appearance and thickness, while also pointing to the merits of rigorous validation studies versus an accepted gold standard [8,9,17]. Thus, future studies should attempt to assess measurement reproducibility (within and between operators) and further profile symptoms and refine exposure characterization (pack years), as well as evaluate agreement with endoscopy/EUS or cross-sectional imaging wherever available, to determine if cervical esophageal wall thickness and layer appearance can be established as reliable imaging biomarkers for smoking-related injury.

This study has multiple strengths. It is to our knowledge the first study to systematically apply sonography to quantify cervical esophagus wall thickness, wall layer discernability, and diameter in smokers vs non-smokers, building directly on established sonographic normals on cervical esophageal anatomy and employing enhanced high-resolution techniques [4,5,7]. The prospective nature of the study and standardized acquisition in multiple planes also adds to its internal consistency. However, multiple limitations must also be acknowledged. These include a relatively young cohort, which limits generalizability; replication studies in older cohorts with increased cumulative exposure are needed. Furthermore, exposure characterization did not account for dose intensity (cigarettes/day, vaping frequency, hookah frequency), and pack-years or equivalent standardized metrics were not captured. This may prevent dose–response inference and should be addressed in future studies as self-reported smoking history could have led to exposure misclassification. The study design limited causal inference; group matching by age and BMI in future studies would strengthen the evidence base. While employing a single operator minimizes variability in technique application, the study has not evaluated for intra-/inter-observer variability. Formal reliability assessment with at least two independent observers is essential before these techniques can be generalized. Finally, the study did not attempt to correlate any potential physiologic significance of the observed findings with symptoms (endoscopy/EUS/histology) [8,9]. Accordingly, the present findings should be regarded as hypothesis-generating, and we propose a program of linked future studies to address the gaps identified above. Specifically, future work should incorporate detailed pack-year quantification with BMI-stratified analyses, correlation with standardized GERD symptom instruments and pH-impedance monitoring, and concurrent validation against endoscopic and histological reference standards, and intra- and inter-observer reproducibility, and transducer/system dependence should be evaluated to establish the methodological and technical accessibility required for routine clinical use.

## 5. Conclusions

High-frequency ultrasound can detect significant smoking-associated structural differences in the cervical esophagus. Compared with non-smokers, habitual smokers demonstrated increased esophageal wall thickness, greater wall layer conspicuity, and larger esophageal diameters, and smoking status remained the dominant independent predictor of wall thickness after adjustment for demographic variables. Smoking duration was positively associated with wall thickness and, more strongly, with the number of sonographically discernible wall layers, supporting a cumulative exposure-related remodeling signal. These findings extend prior work establishing the feasibility and anatomic basis of cervical esophageal sonography and suggest that transcutaneous ultrasound may provide a practical, non-invasive approach for research phenotyping of smoking-related esophageal change. Larger studies incorporating refined exposure metrics (including daily consumption and pack-years), sex and BMI stratified analyses, symptom correlation, reproducibility testing with inter-observer reliability assessment, and validation against endoscopy/EUS or cross-sectional imaging are warranted to define the clinical and biological significance of these ultrasound markers. Diagnostic performance evaluation (ROC analysis, sensitivity/specificity, and clinically meaningful thresholds) should be incorporated in future adequately powered studies. The present results should therefore be interpreted as preliminary imaging evidence of a smoking–esophageal structural association rather than a clinically actionable finding. Further methodological refinement, statistical validation, and external verification—including pack-year quantification, BMI-stratified analyses, GERD symptom correlation, and agreement with endoscopic and histological reference standards—will be required before these sonographic markers can be considered for incorporation into clinical guidelines.

## Figures and Tables

**Table 1:** Comparison of demographic and cervical esophageal ultrasound measurements between smokers and non-smokers, including percentage differences relative to non-smokers.

Variable	Non-Smokers (Mean ± SD)	Smokers (Mean ± SD)	Percentage of Difference	t-Value	*p*-Value	Cohen’s d
Age (years)	23.53 ± 1.48	26.90 ± 3.95	14.3%	4.37	<0.001	1.13
Height (cm)	173.87 ± 4.01	176.13 ± 5.39	1.3%	1.85	0.070	0.48
Weight (kg)	76.13 ± 13.06	89.47 ± 12.08	17.5%	4.11	<0.001	1.06
BMI	25.2 ± 4.40	28.84 ± 3.83	14.4%	3.41	0.001	0.88
Wall Thickness (mm)	2.61 ± 0.23	3.06 ± 0.25	19.2%	7.05	<0.001	1.82
Number of Wall Layers	3.60 ± 0.62	5.03 ± 0.76	39.7%	7.97	<0.001	2.06
Transverse Diameter (mm)	7.87 ± 0.34	11.68 ± 2.56	50.0%	8.25	<0.001	2.13
Longitudinal Diameter (mm)	8.26 ± 0.38	12.90 ± 3.14	55.4%	8.03	<0.001	2.07

**Table 2:** Multiple linear regression analysis of factors associated with esophageal wall thickness (mm).

Predictor	B (mm)	95% CI for B	SE	β	t	*p*
Intercept	2.350	1.64 to 3.06	0.352	–	6.668	<0.001
Age (years)	0.014	−0.008 to 0.036	0.011	0.146	1.294	0.201
Weight (kg)	−0.0009	−0.0069 to 0.0051	0.003	−0.037	−0.336	0.738
Smoking status (smoker) *	0.411	0.24 to 0.58	0.084	–	4.911	<0.001

Model summary: R = 0.693; R^2^ = 0.480; adjusted R^2^ = 0.452; RMSE = 0.245; F (3.56) = 17.23, *p* < 0.001; *N* = 60. * Non-smoker is the reference category. B = unstandardized regression coefficient; SE = standard error; β = standardized coefficient (reported only for continuous predictors).

**Table 3:** Cervical esophageal ultrasound measurements among smokers by smoking type.

Smoking Type	*n*	Wall Thickness (mm), Mean ± SD	Number of Wall Layers, Mean ± SD	Transverse Diameter (mm), Mean ± SD	Longitudinal Diameter (mm), Mean ± SD
Cigarettes	9	3.03 ± 0.24	4.56 ± 0.53	11.24 ± 2.42	12.66 ± 3.32
Vaporizers	2	2.90 ± 0.00	4.50 ± 0.71	9.00 ± 0.14	9.35 ± 0.50
Vaporizers & cigarettes	19	3.10 ± 0.27	5.32 ± 0.75	12.17 ± 2.61	13.39 ± 3.05

ANOVA: wall thickness F (2.27) = 0.60, *p* = 0.557; number of wall layers F (2.27) = 4.36, *p* = 0.023; transverse diameter F (2.27) = 1.64, *p* = 0.213; longitudinal diameter F (2.27) = 1.60, *p* = 0.220.

## Data Availability

The datasets used during the current study are available from the corresponding author on reasonable request.

## Abbreviations

The following abbreviations are used in this manuscript:

ANOVA	Analysis of Variance
ACG	American College of Gastroenterology
B-mode	Brightness mode
BMI	Body Mass Index
CI	Confidence Interval
CT	Computed Tomography
EoE	Eosinophilic Esophagitis
EUS	Endoscopic Ultrasound/Endoscopic Ultrasonography
GERD	Gastroesophageal Reflux Disease
LES	Lower Esophageal Sphincter
LUS	Longitudinal Ultrasound Scan/Longitudinal plane
